# Light induced assembly and self-sorting of silica microparticles

**DOI:** 10.1038/s41598-018-19282-5

**Published:** 2018-01-19

**Authors:** Neus Vilanova, Isja de Feijter, Abraham J. P. Teunissen, Ilja K. Voets

**Affiliations:** 1 Laboratory of Macromolecular and Organic Chemistry, Department of Chemical Engineering and Chemistry, Eindhoven University of Technology, P.O. Box 513, 5600 MD Eindhoven, The Netherlands; 2 Laboratory of Physical Chemistry, Department of Chemical Engineering and Chemistry, Eindhoven University of Technology, P.O. Box 513, 5600 MD Eindhoven, The Netherlands; 3 Institute for Complex Molecular Systems, Eindhoven University of Technology, Post Office Box 513, 5600 MD Eindhoven, The Netherlands; 4 Present Address: SAXSLAB, Diplomvej 377, 2800 Kgs Lyngby, Denmark

## Abstract

To tailor the properties of colloidal materials, precise control over the self-assembly of their constituents is a prerequisite. Here, we govern the assembly of silica particles by functionalization with supramolecular moieties which interact with each other via directional and reversible hydrogen bonding. Through a generally applicable synthesis protocol, two different types of self-complementary hydrogen bonding moieties, BTA- and UPy-derivatives, are anchored to silica particles. Their self-assembly is initiated by the UV-induced removal of a photolabile protecting group, allowing the formation of hydrogen bonds between tethered molecules. The light-induced assembly of BTA- and UPy-decorated colloids in single-component dispersions and colloidal self-sorting in mixed dispersions is studied. Furthermore, we demonstrate that UPy-colloids can dissasemble upon addition of traces of a competitive binder (NaPy). This work provides further insight into the utility of supramolecular handles to orchestrate the assembly of micron-sized colloids via non-oligonucleotide hydrogen-bonding units.

## Introduction

The ability to control molecular self-assembly via non-covalent interactions is an emerging bottom-up strategy to create complex metamaterials. In turn, well-defined molecular recognition can be used as a powerful tool to direct the assembly of micron-sized^1–3^ colloids or even macroscopic objects^4^ and create larger aggregated structures with emergent optical^5–7^, mechanical^8^ or catalytic properties^9,10^. Molecular control over the assembly of micron-sized particles can be realized through particle surface-grafting of molecules exhibiting dipole-dipole^11,12^, metal coordination^13^, hydrophobic forces^14,15^ and/or hydrogen bonding (H-bonding)^16–18^. Ideally, the tethered motifs should be easy to synthesize, responsive, externally addressable and provide directional, dynamic and specific bonding within the assemblies. Moreover, to facilitate colloidal assembly, a precise control between the interplay of non-specific surface forces (attractive and/or repulsive) is crucial^19^.

A popular strategy in the field is the functionalization of colloids with complementary DNA strands^20,21^. Great advances in insight and structural complexity have been achieved in the last 20 years, enabling the assembly of colloids into both disordered and exotic ordered states^22,23^, in a reversible^22,24,25^ or irreversible^26^ manner and at the nano-^27,28^ and mesoscale^25,29^. Notwithstanding, DNA-technology has its limitations, such as high cost and restricted environmental conditions^27,30^ because the strands only assemble in water at sufficiently high ionic strengths and at low temperature, precluding material synthesis and processing from organic solvents. Furthermore, up to now, only temperature has been used as external trigger of assembly, while recent advances towards light sensitive DNA-strands can be utilized in future to develop dual-responsive systems sensitive to both temperature and light^31^. A promising alternative strategy exploits the potential of synthetic self-assembling molecules to fabricate multiresponsive functional materials. The incorporation of photoresponsive motifs, such as azobenzenes for example, has been proposed to guide particle assembly at the nano- and mesoscale^5,11,32^. Azobenzenes can be reversibly switched between two isomers, only one of which has a significant dipole moment and thus gives rise to intermolecular dipolar interactions. Interestingly, the behaviour of the azobenzenes is tuneable by molecular design; i.e. modification of the substituents in the aromatic ring. In this manner one can modulate which isomer (cis or trans) has a large dipole moment and (red) shift the wavelength of photo-isomerization^33,34^. Disadvantageously, however the induced dipole-dipole interactions are not specific. Azobenzene-mediated colloidal assembly can however be made selective, when used in combination with cyclodextrins^15,35,36^.

**Figure 1:**
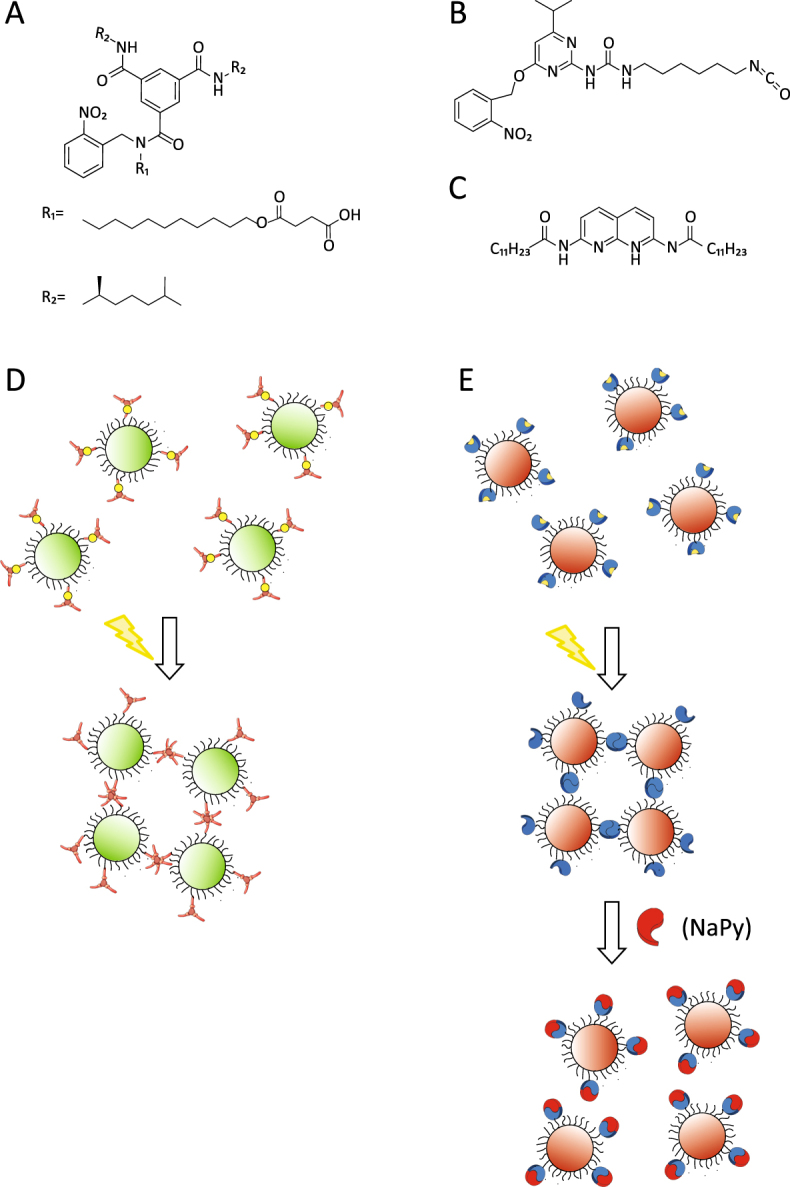
Chemical structures of the (**A**) BTA-, (**B**) UPy- and (**C**) NaPy derivatives. Schematic representation of the behavior of (**D**) BTA- and E) UPy-colloids. Upon UV-irradiation, the labile *o*-nitrobenzyl group is cleaved off and the H-bonds become active, triggering colloidal clustering. (**E**) Addition of NaPy disrupts clusters of UPy-colloids.

Aiming to explore the possibilities and limitations of molecular control over colloidal assembly further, we here introduce a versatile route, built upon a previous work^16^, towards light-activated selective assembly of silica microparticles via H-bonding. We select H-bonding for colloidal assembly, since it is specific, directional and tuneable in strength. We utilize two different types of photo-activatable motifs that interact through H-bonds in an orthogonal fashion (i.e., exclusively with the same motif) to prepare dual-responsive silica microparticles, sensitive to light and temperature. Here we coat silica microparticles with either o-nitrobenzyl protected benzene-1,3,5-tricarboxiamide (BTA) derivatives or *o*-nitrobenzyl protected 2-ureido-4[1 H]-pyrimidinone (UPy) derivatives (Fig. 1) via a mild coupling reaction. Both BTAs and UPys motifs can readily be functionalized with a variety of side chains for easy coupling reactions to the colloids^37^, enhanced solubility in aqueous^17^ and organic media and thermo- and light-responsiveness^16^. When molecularly dissolved in a mixed solution, these motifs preferentially self-assemble in organic media of low polarity via H-bonds into single component stacks and homodimers, respectively^38^, this phenomenon is so-called narcissistic aggregation. We profit from this specific selectivity at the molecular level, to prepare two-component dispersions of silica microparticles, which spontaneously self-sort into single-specie clusters upon UV-illumination, hence increasing the generality and modularity of our approach. Interestingly, colloidal gels with this arrested complex architecture^7,37–40^ and other similar illustrative examples^33,41^ have shown promising mechanical properties and could serve as a model to understand biological phenomena. The exploitation of our approach based on the high specificity and fidelity of H-bonding supramolecular moieties, has resulted for the first time in a light-activated colloidal self-sorting of identical particles into single-species clusters.

In summary, two types of supramolecular colloids, BTA- and UPy-colloids, were designed and synthesized to photo-initiate colloidal assembly in single component dispersions and colloidal self-sorting in mixed dispersions. These results demonstrate that the intrinsically responsive nature of small molecules can be harnessed effectively to program the mesoscale assembly of silica microparticles whose properties can be dynamically modulated, which is an essential stepping stone for the bottom-up manufacturing of complex colloidal metamaterials in aqueous and organic milieu.

## Results and Discussion

### Efficient coupling of supramolecular moieties under mild conditions

**Figure 2:**
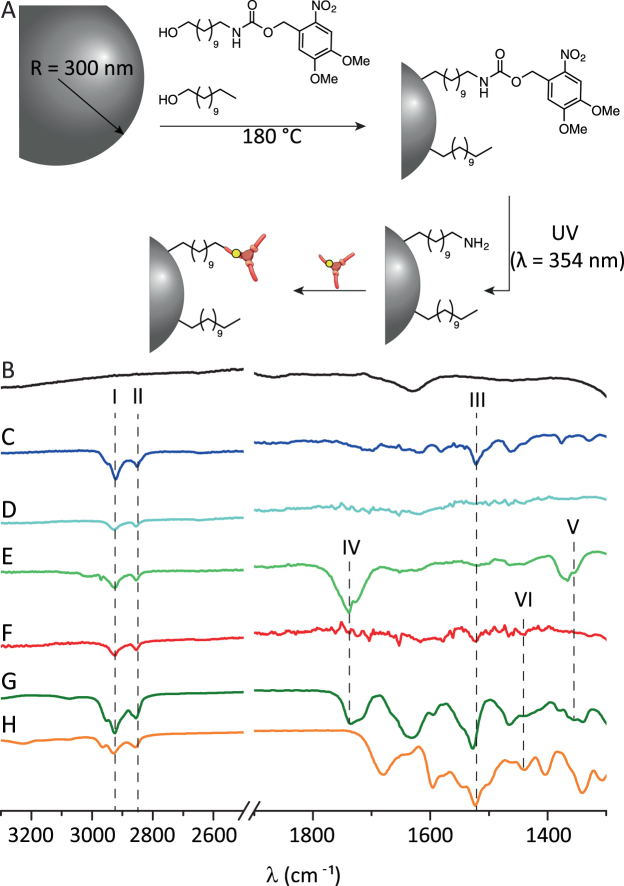
(**A**) Representative scheme of the functionalization of particles with BTA. Spectra of silica colloids at the different functionalization steps: (**B**) bare silica colloids, (**C**) NVOC-C_11_- functionalized, (**D**) amine- functionalized, (**E**) BTA- functionalized and (**F**) UPy-functionalized colloids. (**G**) BTA and (**H**) UPy molecules before coupling to the colloids.

To regulate colloidal assembly via supramolecular chemistry, a mild, efficient and generally applicable two-step coupling strategy was developed. Herein, hydrophobic silica colloids are first produced and subsequently post-functionalized with the supramolecular moieties at room temperature, thus preventing molecular degradation (Fig. 2A). The two-step functionalization begins with a simultaneous coupling of stearyl alcohol (a C_18_ spacer) and a NVOC-C_11_-OH linker to the particles. Next, the NVOC group is cleaved by light (λ_max_ = 354 mm) from the linker to afford primary amines for an efficient coupling of various functional groups under mild reaction conditions, like *o*-nitrobenzyl protected benzene-1,3,5-tricarboxamide (BTA) and *o*-nitrobenzyl protected 2-ureido-4[1 H]-pyrimidinone (UPy) derivatives. The stearyl alcohol plays several important roles. First it makes the particles hydrophobic, allowing them to be dispersed in organic solvents. Second, it enhances colloidal stability since it acts as a steric barrier inhibiting direct contact between two or more particles. And lastly, it suppresses the attractive Van der Waals interactions by decreasing the refractive index mismatch between the colloids and the solvent^37^. This classical colloidal almost hard-sphere-like model system of stearyl alcohol coated silica beads in cyclohexane, thus serves as an excellent starting point for supramolecular control over colloidal assembly, as other attractions are effectively minimized.

The different functionalization steps were monitored by infrared spectroscopy (IR) on small functionalized colloids (*D* = 26 nm) to maximize the intensity of the peaks corresponding to vibrations of the different tethered moieties. Figure 2 shows the spectra of plain (B), NVOC-C_11_- (C) and amine-functionalized (D) colloids together with the spectra of the two final products, i.e. supramolecular BTA- (E) and UPy-colloids (F). As a reference, the spectra of the supramolecular moieties prior to attachment are also shown (Fig. 2G,H). The peaks at 2920 (I) and 2850 (II) cm^−1^ present in all the spectra of functionalized particles are mainly attributed to the stretching bands of the -CH_2_ and -CH_3_ groups of the alkyl chains. NVOC-C_11_-functionalized colloids (C) exhibit a peak at 1520 cm^−1^ (III), which corresponds to the stretching band of the N-O group from the NVOC functional group. The lack of this band in the IR spectrum of the washed deprotected colloids (D), together with the appearance of small peaks around 1550 cm^−1^, corresponding to the stretching band of primary amines, suggested that the NVOC group was efficiently cleaved off, hence yielding the desired amine-functionalized particles, onto which supramolecular moieties can be coupled. As expected, the spectra of BTA-functionalized colloids (E) and the BTA moiety (G) bear strong similarities, displaying broad peaks of the C=O and C-N stretching bands at 1740 (IV) and 1370 (V) cm^−1^, respectively. In addition, the N-O peak III from the protecting *o*-nitrobenzyl group was also observed. Similarly, UPy-colloids display, although weak, the bands at 1520 (III), 1740 (IV) and 1445 (VI) cm^−1^ from the N-O, C=O and -CS-NH of the functional groups, respectively, also present in the spectrum of the UPy moiety (H). Thus the IR results indicate successful coupling of both molecules onto the silica colloids as the IR spectra taken during the synthesis of the supramolecular colloids display the characteristic vibrations of the starting (Fig. 2B,C,G,H), intermediate (Fig. 2D) and final product (Fig. 2E,F) of the reaction.

**Figure 3:**
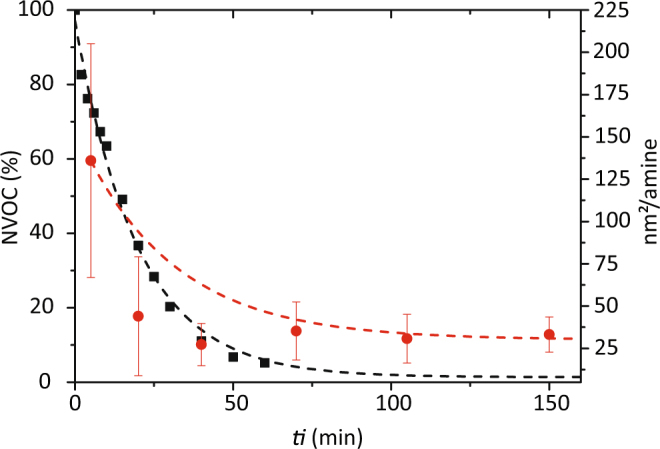
Conversion of the deprotection of the (■) NVOC-C_11_-OH linker in solution (expressed as the percentage of the NVOC group present) and () on the colloids (expressed as the surface area per amine on the colloids) as a function of the irradiation time (*t*_*i*_). Lines are drawn to guide the eye.

To validate the IR results and estimate the maximum number of BTA and UPy molecules that can be coupled to the colloids, the number of primary amines present onto small colloids during the deprotection step was quantified using a UV-spectroscopic essay for bioconjugation as described in the Methods section. Figure 3 shows the evolution of the surface area per amine on the colloids as a function of the deprotection time (*t*_*i*_). After 60 min of deprotection, a plateau was reached at approximately 46.4 nm^2^/amine, indicating that the deprotection of the NVOC group onto the colloids was complete. This density corresponds to approximately 24350 amines per large colloid (*D* = 600 nm)^37^, which in turn equals the maximal number of supramolecular moieties that can be coupled to the particles.

A parallel kinetic study was carried out for the NVOC-C_11_-OH linker free in solution and a similar pattern was observed. After 60 min of irradiation, 95% of the NVOC groups were converted to amines (Fig. 3).

### Self-assembly of the silica colloids

**Figure 4:**
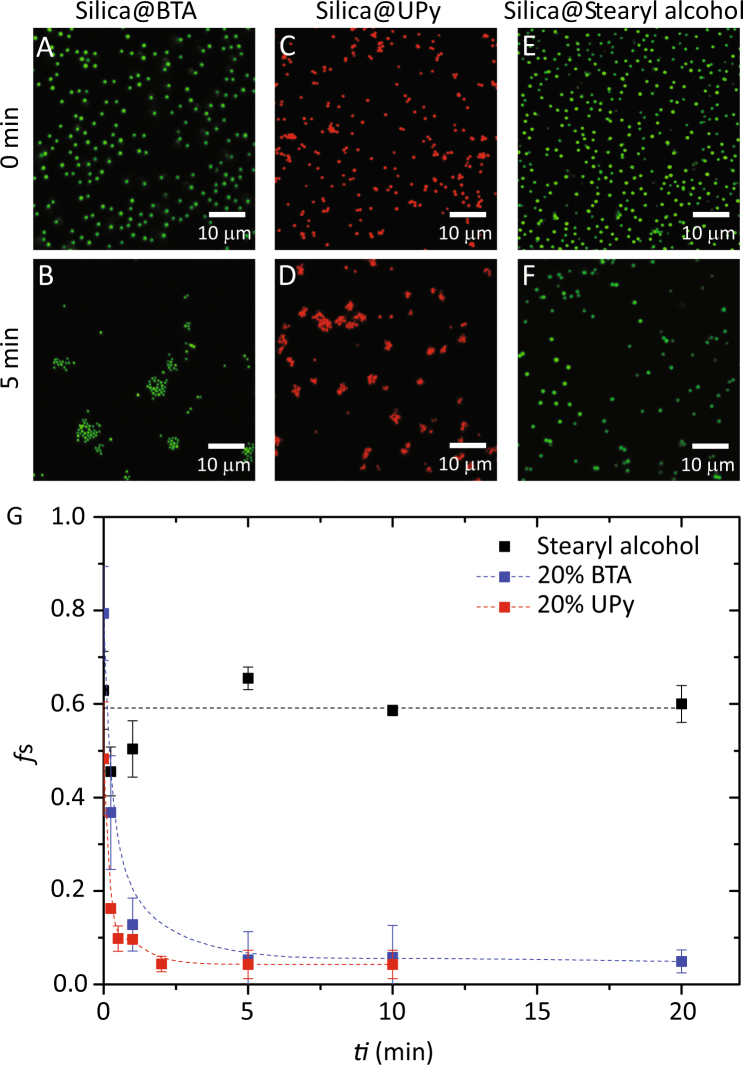
Confocal images of BTA- (**A**,**B**) UPy- (**C**,**D**) and stearyl-coated (**E**,**F**) colloids before (**A**,**C**,**E**) and after 5 min of UV-irradiation (**B**,**D**,**F**). (**G**) Fraction of singlets (*f*_s_) as a function of UV-irradiation (*t*_*i*_) (λ_max_ = 354 mm) for BTA-, UPy- and stearyl-coated colloids. Lines are drawn to guide the eye.

To confirm that the H-bonds from the tethered molecules can be activated by light, through cleavage of the *o*-nitrobenzyl protective group, the assembly of BTA- and UPy-colloids was studied by means of confocal microscopy as a function of the irradiation time, *t*_*i*_. The fraction of singlets (*f*_*s*_) was determined by quantitative image analysis as previously described^37^. Before irradiation (*t*_*i*_ = 0 min) most of the colloids were nicely dispersed as singlets (Fig. 4A,C and E) with *f*_*s*_ = 0.63, 0.80 and 0.48 for stearyl alcohol coated particles, BTA-colloids, and UPy-colloids respectively. This confirms on the one hand, that the Van der Waals forces are weak (estimated to be about −0.2 k_B_T at contact), and on the other hand that the *o*-nitrobenzyl protecting group effectively blocks H-bond formation prior to irradiation. Notice the somewhat smaller fraction of singlets prior to illumination for UPy-colloids relative to BTA-colloids (*f*_*s*_ = 0.48 vs. 0.80), which we tentatively attribute to a less effective inactivation of H-bonds by the *o*-nitrobenzyl and limited solubility of the UPy derivative in cyclohexane. Upon irradiation, *f*_*s*_ decreases drastically within 5 minutes to a vanishingly small value of *f*_*s*_ ~0.05 for both BTA- and UPy-colloids (Fig. 4B,D,G, see Figure S3 for more exemplary pictures). Conversely, as expected, the fraction of singlets is independent of irradiation time for stearyl alcohol coated particles (Fig. 4E–G), which are not UV-light responsive. These results clearly demonstrate that the attractive hydrogen bonding interactions between tethered supramolecular moieties can be effectively shielded prior to activation by UV-light, which triggers colloidal assembly as a consequence of the dominant intermolecular association.

### Dissolution of UPy clusters upon addition of a hetero-complementary motif

**Figure 5:**
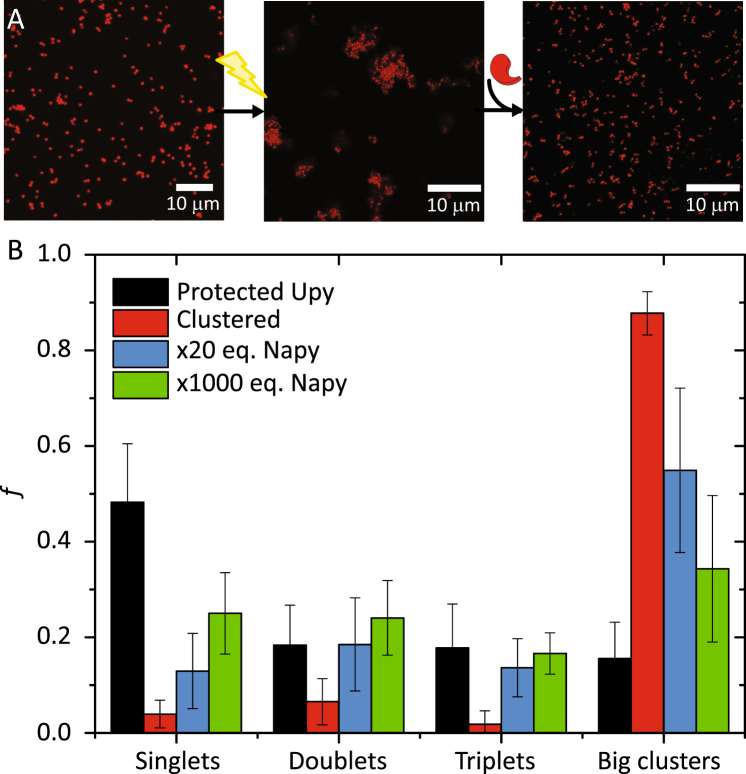
Evolution of UPy-colloids upon UV-irradiation (λ_max_ = 354 mm) and addition of free 20 and 1000 equivalents of NaPy (**A**). Fraction (*f*_*s*_) of singlets, doublets, triplets and big clusters (formed by more than 4 particles) upon irradiation and addition of NaPy (**B**).

In the above, we have shown that colloidal assembly can be photo-initiated via *o*-nitrobenzyl protected BTA and UPy derivatives. In a previous work, we demonstrated that the association strength between BTA-colloids can be controlled by temperature^16^. Here, we explore the use of an external, competitive binder to control the assembly of UPy-colloids. UPy homodimers can be dissolved upon addition of a complementary supramolecular moiety, a 2,7-diamido-1,8-naphthyridines derivative (NaPy), yielding hence UPy-NaPy heterodimers^42^. Self-assembly is favoured at low NaPy concentrations, while heterodimerization is predominant at high concentrations^43^. Hence, in principle UPy colloidal clusters can be selectively disrupted upon the addition of the competitive binder NaPy. Based on this, free NaPy was added to a dispersion of UPy colloids in cyclohexane after 20 min of irradiation to test by confocal microscopy whether NaPy can be utilized to break-up clusters of UPy-functionalized colloids (Fig. 5). As expected, light-induced assembly of UPy-colloids was evidenced by a dramatic reduction of the singlet fraction from *f*_*s*_ = 0.48 to 0.04 after 20 min illumination and a concomitant increase in the abundance of small and especially large clusters. Addition of a 20-fold excess of NaPy broke these clusters apart, albeit partially, resulting in a singlet fraction *f*_*s*_ = 0.13, i. e. substantially lower than prior to UV-illumination. Addition of a larger, 1000-fold excess of NaPy disrupted more clusters and further increased the singlet fraction to *f*_*s*_ = 0.25. However, a full recovery to the initial *f*_*s*_ = 0.48 (without simultaneously raising the sample temperature) was not attainable, since this 1000-fold excess corresponds to a NaPy concentration near the solubility limit in cyclohexane.

### Engineering orthogonality in the colloidal domain

**Figure 6:**
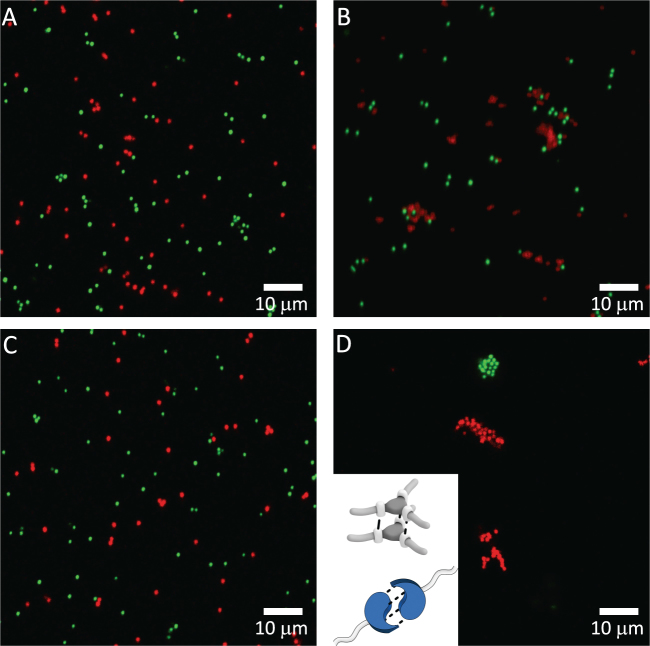
Confocal images of a mix of stearyl-coated colloids (green) and BTA-colloids (red) (**A**) before and (**B**) after 30 min irradiation. Confocal images of an equal mixture of BTA-colloids (green) and UPy-colloids (red) (**C**) before and (**D**) after 30 min of irradiation (λ_max_ = 354 mm). Inset: scheme of the triple and quadruple H-bonding between BTAs and UPys, where the dashed lines represent the H-bonds between the moieties.

Aiming to investigate whether supramolecular motifs can be used to create self-sorting colloids, we explored the behavior of binary mixtures of supramolecular colloids. As a preliminary test of selectivity, a 0.1 wt% dispersion of stearyl alcohol coated colloids (green) and a 0.1 wt% dispersion of protected BTA-colloids (red) were mixed in equal volumes. Before illumination, most of the colloids were present as singlets (Fig. 6A). Upon irradiation, stearyl alcohol functionalized colloids remained dispersed, while the red BTA-colloids formed clusters (Fig. 6B). This selective assembly again corroborates that H-bonds between supramolecular BTA-colloids are the dominant attractive interactions. To assess whether the orthogonality of the triple- and quadruple H-bonding motifs translates into the colloidal domain, protected BTA-(green) and UPy-colloids (red) were mixed following the same protocol. Once more, most colloids remained in a non-aggregated state prior illumination (Fig. 6C). After irradiation, most of the colloids nicely assembled in an orthogonal fashion, resulting in two types of clusters comprising either BTA or UPy colloids (Fig. 6D, see Figure S4 for more exemplary pictures). These results evidence that the orthogonality of the supramolecular interactions at the molecular level can be effectively transferred to the colloidal domain, introducing a useful tool to build mixed systems with controlled aggregation behavior.

## Conclusions

Herein we introduce a simple and modular approach to achieve reversible^16^ and controlled self-assembly of micron-sized colloids by surface-anchoring of supramolecular moieties, such as BTA and UPy derivatives. As the behaviour of the colloids is governed by light-responsive tethered motifs, their self-assembly can be externally tuned. Upon deprotection with UV-light, supramolecular moieties are ‘activated’, thereby initiating colloidal self-organization via H-bonding. Clusters of UPy-colloids could be dissolved partially by addition of free NaPy derivatives and colloidal self-sorting into single-species clusters was realized in binary mixtures of BTA- and UPy-colloids due to the orthogonality of the non-covalent intermolecular interactions. These findings demonstrate the utility of small molecular guides to control colloidal assembly, which we will exploit in the future to build photo-sensitive gels and Pickering emulsions.

## Experimental

### Materials

The synthesis of the supramolecular moieties is described in the Supplementary information. Solvents were dried over molsieves prior to use.

#### Synthesis of the core-shell particles

Based on the Stöber method, 600 nm in diameter core-shell particles with a fluorescein isothiocyanate (FITC) core were synthesized according to ref.^37^. The same protocol was followed to synthesize particles with a core containing the red dye Rhodamine B isothiocyanate (RITC). Throughout the text these are referred to as green (FITC) and red (RITC) colloids.

### Functionalization of core-shell particles

- Synthesis of amine-functionalized particles: a detailed description of the synthesis of NVOC-C_11_- and stearyl alcohol-functionalized particles with a 20/80 molar ratio has already been reported^37^. The amine-functionalized particles were obtained upon cleavage of the NVOC group as follows. A dispersion of the NVOC-functionalized particles in chloroform was irradiated with a UV-oven (Luzchem LZC-4V UV oven equipped with 8 × 8 Watt UV-A light bulbs with λ_max_ = 354 mm) for 1 hour under continuous stirring at room temperature. After illumination, the resulting amine-functionalized particles were washed 6 times with chloroform.

- BTA-functionalized particles: 20 mg of green amine-functionalized particles were dispersed in 3 mL of dry chloroform, to this suspension BTA derivative (18 mg, 0.02 mmol), (Benzotriazol-1-yloxy)tripyrrolidinophosphonium hexafluorophosphate (PyBOP, 10.4 mg, 0.02 mmol) and N,N-Diisopropylethylamine (DIPEA, 17.4 μl, 0.1 mmol) were added. The dispersion was stirred for 16 hours at room temperature under an argon atmosphere. Afterwards, particles were washed 6 times with chloroform and dried overnight *in vacuo* at 70 °C.

- UPy-functionalized particles: 20 mg of red amine-functionalized particles were dispersed in 3 mL of dry chloroform together with UPy derivative (18.2 mg, 0.04 mmol). The suspension was stirred for 16 hours at room temperature under an argon atmosphere. Afterwards, particles were washed 6 times with chloroform and dried overnight *in vacuo* at 70 °C.

- Functionalization of commercial Ludox AS-40 particles: small commercial particles of 26 nm in diameter were used for the IR characterization and determination of the deprotection kinetics of the NVOC group on the particles. Their functionalization was carried out as in above-described for the core-shell particles. For the IR experiments, Ludox AS-40 particles were fully functionalized with the NVOC-C_11_-OH linker only (i.e. no stearyl alcohol) to enhance the intensity of the peaks coming from the different functional groups by maximizing the organic/inorganic matter ratio.

## Methods

### IR spectroscopy

Functionalization of the colloids at the different steps of the protocol was confirmed by IR. IR spectra of the colloids in the solid state were obtained using a Perkin-Elmer spectrum equipped with a UATR Two sample stage. Over 40 spectra were recorded for each sample.

### UV-Vis spectroscopy

To probe the deprotection of the NVOC group onto the colloids, several vials with 20 mg each of small NVOC-C_11_-colloids dispersed in CHCl_3_ were irradiated within a UV-oven (Luzchem LZC-4V UV equipped with 8 × 8 Watt UV-A light bulbs with λ_max_ = 354 mm) for different times. While deprotecting, the dispersions were gently stirred with a magnetic stirrer to avoid sedimentation and keep the surface of the colloids always exposed to the light, ensuring a homogenous deprotection and hence, an homogenous amine coverage. The kinetics of deprotection were followed by monitoring the primary amines onto the particles as a function of the exposure times. To do so, a commonly used UV-spectroscopic essay for bioconjugation was chosen and adapted, see reference^44^ for a detailed description. Briefly, succinimidyl 3-(2-pyridyldithio)propionate (SPDP) molecules were coupled to the amines onto the particles, followed by the cleaving of the UV-active pyridine-2-thione upon the addition of 1,4-Dithiothreitol (DTT). Assuming that all reactions take place, the number of pyridine-2-thiones cleaved corresponds to the number of amines present. Note that the protocol was verified measuring the number of amines from amine-functionalized commercial particles. All experiments were carried out in duplicate.

### Confocal microscopy

A Nikon Ti Eclipse laser scanning confocal microscope was used to characterize the clustering process of the supramolecular colloids. Microscopy samples were prepared by dispersing 0.1 wt% of the supramolecular colloids in cyclohexane and sonicate for 20 min. Afterwards the dispersion was irradiated in a UV oven (Luzchem LZC-4V UV equipped with 8 × 8 Watt UV-A light bulbs with λ_max_ = 354 mm). At different deprotecting times, a 25 μl aliquot was withdrawn and placed onto a slide using a spacer (Grace BioLabs Secure seal imaging spacer, Sigma-Aldrich). Tens of images (accounting for hundreds of particles) were analyzed by ImageJ and Matlab according to the protocol described in ref.^37^.

## Electronic supplementary material

Supplementary information

## References

[1] Cantekin S, De Greef TFA, Palmans ARA (2012). Benzene-1,3,5-tricarboxamide: A versatile ordering moiety for supramolecular chemistry. Chem. Soc. Rev..

[2] Schacher FH, Rupar PA, Manners I (2012). Functional block copolymers: Nanostructured materials with emerging applications. Angew. Chemie - Int. Ed..

[3] Elacqua E, Lye DS, Weck M (2014). Engineering orthogonality in supramolecular polymers: From simple scaffolds to complex materials. Acc. Chem. Res..

[4] Harada A, Kobayashi R, Takashima Y, Hashidzume A, Yamaguchi H (2011). Macroscopic self-assembly through molecular recognition. Nat. Chem..

[5] Klajn R, Wesson PJ, Bishop KJM, Grzybowski BA (2009). Writing self-erasing images using metastable nanoparticle ‘inks’. Angew. Chemie - Int. Ed..

[6] Hu C, West KR, Scherman OA (2016). Hollow mesoporous raspberry-like colloids with removable caps as photoresponsive nanocontainers. Nanoscale.

[7] Gentili, D., Ori, G., Ortolani, L., Morandi, V. & Cavallini, M. Cooperative and Reversible Anisotropic Assembly of Gold Nanoparticles by Modulation of Noncovalent Interparticle Interactions. *ChemNanoMat*10.1002/cnma.201700212 (2017).

[8] Di Michele, L. *et al*. Aggregation dynamics, structure, and mechanical properties of bigels. *Soft Matter* **10** (2014). 10.1039/c3sm52558a 24668413

[9] Chen Z (2014). Light controlled reversible inversion of nanophosphor-stabilized pickering emulsions for biphasic enantioselective biocatalysis. J. Am. Chem. Soc..

[10] Zhao H (2016). Reversible trapping and reaction acceleration within dynamically self-assembling nanoflasks. Nat. Nanotechnol..

[11] Klajn R, Bishop KJM, Grzybowski BA (2007). Light-controlled self-assembly of reversible and irreversible nanoparticle suprastructures. Proc. Natl. Acad. Sci. USA.

[12] Chovnik, O., Balgley, R., Goldman, J. R. & Klajn, R. Dynamically self-assembling carriers enable guiding of diamagnetic particles by weak magnets. *J. Am. Chem. Soc*. **134** (2012). 10.1021/ja309633v 23181449

[13] Wang Y (2013). Patchy particle self-assembly via metal coordination. J. Am. Chem. Soc..

[14] Chen Q, Bae SC, Granick S (2011). Directed self-assembly of a colloidal kagome lattice. Nature.

[15] Han K, Go D, Hoenders D, Kuehne AJC, Walther A (2017). Switchable supracolloidal coassembly of microgels mediated by host/guest interactions. ACS Macro Lett..

[16] De Feijter, I., Albertazzi, L., Palmans, A. R. A. & Voets, I. K. Stimuli-responsive colloidal assembly driven by surface-grafted supramolecular moieties. *Langmuir* **31** (2015). 10.1021/la5031872 25489659

[17] Van Ravensteijn BGP, Vilanova N, De Feijter I, Kegel WK, Voets IK (2017). Temperature-Induced, Selective Assembly of Supramolecular Colloids in Water. ACS Omega.

[18] Celiz, A. D., Lee, T.-C. & Scherman, O. A. Polymer-mediated dispersion of cold nanoparticles: using supramolecular moieties on the periphery. *Adv. Mater*. **21** (2009).

[19] Gerth M, Voets IK (2017). Molecular control over colloidal assembly. Chem. Commun..

[20] Mirkin CA, Letsinger RL, Mucic RC, Storhoff JJ (1996). A DNA-based method for rationally assembling nanoparticles into macroscopic materials. Nature.

[21] Storhoff JJ, Mirkin CA (1999). Programmed Materials Synthesis with DNA. Chem. Rev..

[22] Wang, Y. *et al*. Crystallization of DNA-coated colloids. *Nat. Commun*. **6** (2015). 10.1038/ncomms8253 PMC4490366 26078020

[23] Kim AJ, Biancaniello PL, Crocker JC (2006). Engineering DNA-mediated colloidal crystallization. Langmuir.

[24] Dreyfus, R. *et al*. Simple quantitative model for the reversible association of DNA coated colloids. *Phys. Rev. Lett*. **102** (2009). 10.1103/PhysRevLett.102.048301 19257481

[25] Valignat M-P, Theodoly O, Crocker JC, Russel WB, Chaikin PM (2005). Reversible self-assembly and directed assembly of DNA-linked micrometer-sized colloids. Proc. Natl. Acad. Sci. USA.

[26] Rogers WB, Manoharan VN (2015). Programming colloidal phase transitions with DNA strand displacement. Science (80-.)..

[27] Jin R, Wu G, Li Z, Mirkin CA, Schatz GC (2003). What controls the melting properties of DNA-linked gold nanoparticle assemblies?. J. Am. Chem. Soc..

[28] Storhoff JJ (2000). What controls the optical properties of DNA-linked gold nanoparticle assemblies?. J. Am. Chem. Soc..

[29] Wang Y (2012). Colloids with valence and specific directional bonding. Nature.

[30] Mognetti BM, Leunissen ME, Frenkel D (2012). Controlling the temperature sensitivity of DNA-mediated colloidal interactions through competing linkages. Soft Matter.

[31] Yan Y, Chen JIL, Ginger DS (2012). Photoswitchable oligonucleotide-modified gold nanoparticles: Controlling hybridization stringency with photon dose. Nano Lett..

[32] Xue C, Gutierrez-Cuevas K, Gao M, Urbas A, Li Q (2013). Photomodulated self-assembly of hydrophobic thiol monolayer-protected gold nanorods and their alignment in thermotropic liquid crystal. J. Phys. Chem. C.

[33] Manna, D., Udayabhaskararao, T., Zhao, H. & Klajn, R. Orthogonal Light-Induced Self-Assembly of Nanoparticles using Differently Substituted Azobenzenes. *Angew. Chemie - Int. Ed*. **54** (2015). 10.1002/anie.201502419 25959725

[34] Bandara HMD, Burdette SC (2012). Photoisomerization in different classes of azobenzene. Chem. Soc. Rev..

[35] Isenbügel K, Gehrke Y, Ritter H (2012). Photo-switchable behavior of azobenzene-dye-modified silica nanoparticles and their assembly with cyclodextrin derivatives. Macromol. Chem. Phys..

[36] Stricker L, Fritz E-C, Peterlechner M, Doltsinis NL, Ravoo BJ (2016). Arylazopyrazoles as Light-Responsive Molecular Switches in Cyclodextrin-Based Supramolecular Systems. J. Am. Chem. Soc..

[37] Vilanova, N., De Feijter, I. & Voets, I. K. Synthesis and characterization of supramolecular colloids. *J. Vis. Exp*. **2016** (2016). 10.3791/53934 PMC4941986 27168201

[38] Mes, T. *et al*. Network formation in an orthogonally self-assembling system. *ACS Macro Lett*. **1** (2012). 10.1021/mz200108a 35578463

[39] Varrato, F. *et al*. Arrested demixing opens route to bigels. *Proc. Natl. Acad. Sci. USA* **109** (2012). 10.1073/pnas.1214971109 PMC3511146 23129616

[40] Di Michele, L. *et al*. Multistep kinetic self-assembly of DNA-coated colloids. *Nat. Commun*. **4** (2013). 10.1038/ncomms3007 23759922

[41] Han K (2017). Social Self-Sorting of Colloidal Families in Co-Assembling Microgel Systems. Angew. Chemie - Int. Ed..

[42] Teunissen, A. J. P., Van Der Haas, R. J. C., Vekemans, J. A. J. M., Palmans, A. R. A. & Meijer, E. W. Scope and limitations of supramolecular autoregulation. *Bull. Chem. Soc. Jpn*. **89** (2016).

[43] Ligthart, G. B. W. L., Ohkawa, H., Sijbesma, R. P. & Meijer, E. W. Complementary quadruple hydrogen bonding in supramolecular copolymers. *J. Am. Chem. Soc*. **127** (2005). 10.1021/ja043555t 15656599

[44] Hermanson, G. T. *Bioconjugate Techniques*. (Elsevier, 2013).

