# Supplementary material for: Light induced assembly and self-sorting of silica microparticles
Source: Sci Rep. 2018 Jan 19;8:1271. doi: 10.1038/s41598-018-19282-5 (PMC5775198; doi:10.1038/s41598-018-19282-5)
Supplement: Supplementary file 1 — Supplementary information [file 41598_2018_19282_MOESM1_ESM.pdf]

Light induced assembly and self-sorting of silica microparticles

Neus Vilanova, [1] *Isja de Feijter,* [1,†] *Abraham J. P. Teunissen,*[1] *Ilja K. Voets*[1,2,*]

[1] Institute for Complex Molecular Systems, Laboratory of Macromolecular and Organic Chemistry, Eindhoven University of Technology, Post Office Box 513, 5600 MD, Eindhoven, The Netherlands

[2] Laboratory of Physical Chemistry, Eindhoven University of Technology, Post Office Box 513, 5600 MD, Eindhoven, The Netherlands

[†] Present address: SAXSLAB, Diplomvej 377, 2800 Kgs Lyngby Denmark.

Synthesis of the NVOC-C_{11}-OH linker (Figure S1). Synthesis of 2- (11-hydroxyundecyl)isoindoline-1,3-dione (**2**): 11-Bromoundecanol (**1**) (10.008g; 39.8 mmol) and potassium phthalimide (9.60 g; 51.8 mmol) were dissolved in dimethylformamide (DMF, 200 mL). The resulting mixture was stirred at 100 ^{o}C overnight. After cooling to room temperature, the formed precipitate was filtered. The filtrate was diluted with ethylacetate (400 ml) and washed with 500 mL of water and followed by 500 mL of saturated KCl. The combined organic layers were dried over $MgSO_4$ and the solvent was evaporated *in vacuo* to yield a white powder (11.36 g, 90 %) which was further used without purification. ^{1}H NMR ($CDCl_3$): δ = 7.84 (dd, 2H, ArH), 7.70 (dd, 2H, ArH), 3.67 (t, 2H, CH_2N), 3.63 (t, 2H, CH_2OH), 1.67 (t, 2H, aliphatic), 1.55 (t, 2H, aliphatic) 1.40-1.21 (m, 14H, aliphatic). ^{13}C NMR ($CDCl_3$): δ = 168.5, 133.8, 132.2, 123.1, 63.1, 38.1, 32.8, 29.5, 29.4, 29.4,29.3, 28.6, 26.8, 25.7 MALDI-TOF-MS calc.: m/z 317.20, found 318.28 [M+H], 340.24 [M+Na].

Synthesis of 11-aminoundecanol (**3**): 2- (11-hydroxyundecyl)isoindoline-1,3-dione (**2**) (200 mg; 0.63mmol) was dissolved in 0.5 mL of tetrahydrofuran (THF), to which 0.5 mL ($1.57 \cdot 10^{-4}$ mmol) hydrazine hydrate was added. The reaction mixture was refluxed overnight. The excess hydrazine and THF were removed *in vacuo*. The residue was dissolved in 5 mL of chloroform and was washed with 5 mL 3M NaOH, 5 mL brine and 5 mL water. The aqueous layer was back extracted once with 5 mL chloroform and the combined organic layers were dried over $MgSO_4$. The solvent was removed *in vacuo* to yield the pure product (112 mg, 95 %). ^{1}H NMR ($CDCl_3$): δ = 3.63 (t, 2H, CH_2OH), 2.68 (t, 2H, CH_2NH_2), 1.67-1.08 (m, 18H, aliphatic). ^{13}C NMR ($CDCl_3$): δ = 63.0, 42.3, 33.9, 32.8, 29.6, 29.6, 29.5, 29.5, 29.4, 26.9, 25.7. MALDI-TOF-MS calc.: m/z 187.19, found 188.49 [M+H].

Synthesis of 4,5-dimethoxy-2-nitrobenzyl(11-hydroxyundecyl) carbamate (**4**): 4,5-Dimethoxy-2-nitrobenzyl chloroformate (NVOC-Cl, 165 mg; 0.6 mmol) was dissolved in 2 mL THF and the resulting mixture was cooled down to 0 ^{o}C. The 11-aminoundecanol (**3**) was dissolved in 5 mL THF and was cooled to 0 ^{o}C. The NVOC-Cl solution was slowly added to the stirring solution of **3** with a syringe and stirred overnight. The reaction mixture was dried *in vacuo*, dissolved in 3 mL THF and precipitated in 10 mL ice-cold water. The precipitate was centrifuged down at 4000 rpm for 10 min to yield the pure product (153 mg, 60 %). ^{1}H NMR ($CDCl_3$): δ = 7.67 (s, 1H, ArH), 6.96 (s, 1H, ArH), 5.48 (s, 2H, $ArCH_2$), 4.80 (broad s, 1H, NH) 3.97 (s, 3H, $ArOCH_3$), 3.95 (s, 3H, $ArOCH_3$), 3.73-3.52 (m, 2H, CH_2OH), 3.41-3.06 (m, 2H, CH_2NH), 1.62-1.41 (m, 4H, aliphatic) 1.41-1.12 (m, 14H, aliphatic). ^{13}C NMR ($CDCl_3$): δ = 155.9, 153.3, 148.0 128.2, 110.3, 108,2 63.4, 63.1, 56.4, 41.2, 32.8, 29.5, 29.5, 29.4, 29.4, 29.2, 26.8, 25.7. MALDI-TOF-MS calc.: m/z 426.24, found 449.2 [M+Na].

Synthesis of 4,5-dimethoxy-2-nitrobenzyl(4,4-diethoxy-9-oxo-3,10-dioxa-8-aza-4-silahenicosan-21-yl)carbamate (**4**) (NVOC-C_{11}-OH linker): 4,5-dimethoxy-2-nitrobenzyl(11-hydroxyundecyl)carbamate (**3**) (153 mg; 0.36 mmol) was dissolved in 10 mL chloroform. 3-(triethoxysilyl)propyl isocyanate (107 mg;

0.43 mmol) and a drop of dibutyltin dilaurate were added and the reaction mixtures was refluxed overnight. After cooling to room temperature, polymer bound tris(2-aminoethyl)amine was added to scavenge the excess isocyanate. The resin was filtered off and washed with chloroform to yield the pure product in quantitative yield after drying *in vacuo*. ^{1}H NMR ($CDCl_3$): δ = 7.71 (s, 1H, ArH), 7.00 (s, 1H, ArH), 5.50 (s, 2H, $ArCH_2$), 4.86 (broad s, 1H, NH) 3.97 (s, 3H, $ArOCH_3$), 3.95 (s, 3H, $ArOCH_3$), 3.82 (q, 6H, $SiOCH_2CH_3$) 3.29-3.04 (m, 2x2H, CH_2NH), 3.41-3.06 (m, 2H, CH_2NH), 1.73-1.47 (m, 6H, aliphatic) 1.46-1.07 (m, 14H, aliphatic), 0.89 (dt, 9H, $SiOCH_2CH_3$), 0.63 (m, 2H, $SiCH_2$). ^{13}C NMR ($CDCl_3$): δ 155.9, 153.3, 149.3, 148.0 128.2, 110.3, 108.2, 75.6, 63.4, 63.1, 58.4, 56.4, 43.3, 41.2, 32.8, 29.5, 29.5, 29.4, 29.4, 29.2, 26.8, 25.7, 18.3, 7.5. MALDI-TOF-MS calc.: m/z 673.36, found 696.4 [M+Na], 712.4 [M+K].

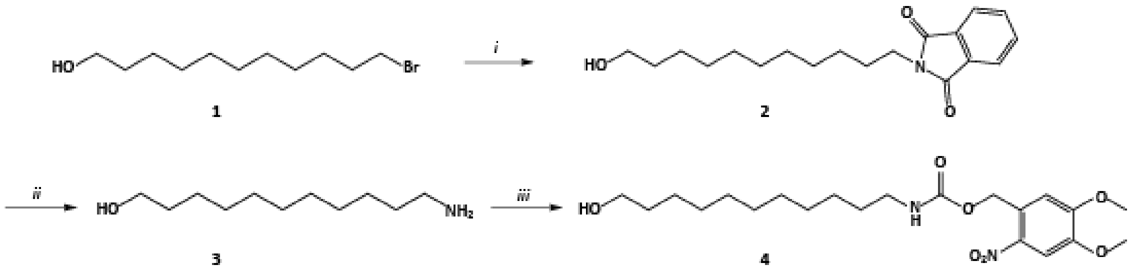

Figure S1. Synthesis of the NVOC-C_{11}-OH linker (**4**). See the description of the full procedure in the experimental section.

Synthesis of carboxylic acid BTA moiety. Hydroxyl-BTA[41] (83 mg; 0.11 mmol) and succinic acid (11.5 mg; 0.12 mmol) were dissolved in 3 mL dry chloroform with trimethylamine (16 μL; 11.6 mg; 0.12 mmol) and a catalytic amount of 4-dimethylaminopyridine. The reaction mixture was refluxed for six hours. The reaction mixture was washed twice with water, the aqueous layers were combined and extracted with chloroform. The organic layers were combined and dried over $MgSO_4$, the solvent was evaporated *in vacuo* and the product (92 mg, 98 %) was used without further purification. ^{1}H NMR (TCE-d2): δ = 8.27 (s, 1H, ArH), 7.92 (d, 1H, ArH), 7.89 (s, 2H, ArH), 7.61 (t, 1H, ArH), 7.42 (m, 1H, ArH), 6.40 (broad s, 2H, NH), 4.92 (s, 2H, NCH_2Ar), 4.01 (t, 2H, $COOCH_2$), 3.45-3.28 (m, 4H, $NHCH_2$), 3.27-3.17 (m, 2H, $NHCH_2$), 2.55 (dt, 4H, $COCH_2CH_2COOH$, 1.68-1.32 (m, 4H, aliphatic), 1.32-0.92 (m, 22H, aliphatic), 0.88 (d, 6H, CH_3), 0.80 (d, 12H $C(CH_3)_2$). MALDI-TOF-MS calc.: m/z 892.59, found 915.58 [M+Na].

Synthesis of UPy-isocyanate. Boc-protected UPy[42] (281 mg; 0.53 mmol) was dissolved in 5 mL of a 1/1 (v/v) mixture of chloroform and trifluoroacetic acid (TFA). The reaction was stirred at room temperature for sixteen hours. The solvent and TFA were removed *in vacuo,* the resulting sticky oil was redissolved in chloroform and toluene and concentrated *in vacuo* three times to remove TFA excess. The residue was dissolved in 10 mL chloroform and washed with 10 mL 1M NaOH and 10 mL brine. The combined organic layers were dried over $MgSO_4$, the solvent was evaporated *in vacuo* and the product was used without further purification (230 mg, 100 %). ^1H NMR ($CDCl_3$): δ = 9.22 (broad s, 1H, NH), 8.12 (d, 1H, ArH), 7.65 (s, 1H, ArH), 7.58-7.43 (m, 1H, ArH), 7.04 (s, 1H, NH), 6.27 (s, 1H, ArH), 5.73 (s, 2H, $ArCH_2O$), 3.35 (q, 2H, $NHCH_2$), 2.86 (h, 1H, $ArCH(CH_3)_2$), 2.67 (t, 2H, NH_2CH_2), 1.66-1.36 (m, 8H, aliphatic), 1.26 (d, 6H, $ArCH(CH_3)_2$).

Synthesis of NaPy. The synthesis of the NaPy moiety is described elsewhere.[43]

Deprotection of NVOC-C_{11}-OH in solution

To study the deprotection kinetics of the NVOC-C_{11}-OH linker free in solution, the molecule was dissolved in deuterated chloroform as this is the solvent where the particles are deprotected. The solution was irradiated with a UV-oven (Luzchem LZC-4V UV equipped with 8x8 Watt UV-A light bulbs with λ_{max}=354 mm) and several NMR spectra were registered at different irradiation times. The conversion of the reaction was determined by integrating the NVOC benzylic protons at δ = 5.5 ppm to primary amines (see Figure S2). NMR spectra were recorded on a 400 MHz NMR (Varian Mercury Vx).

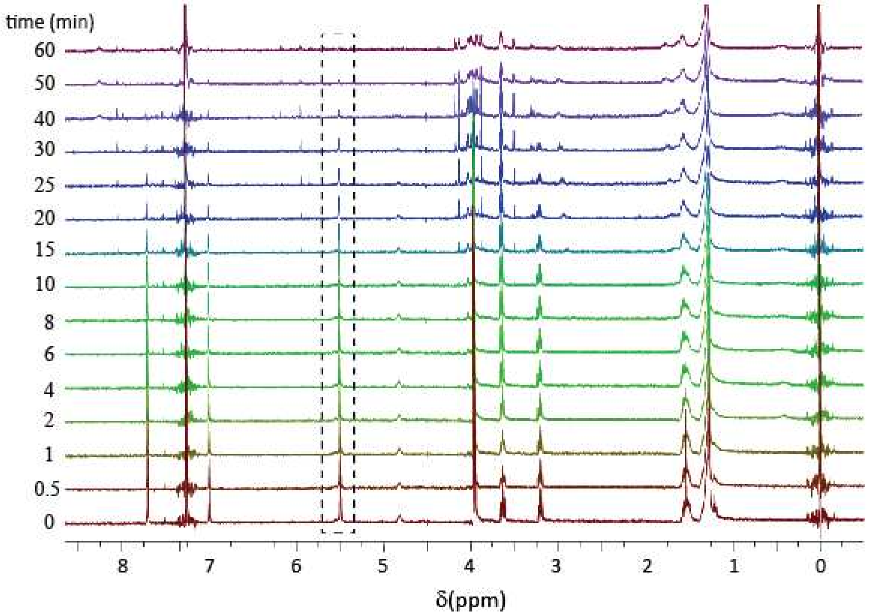

Figure S2. ^{1}H NMR spectra in $CDCl_3$ of the NVOC-C_{11}-linker at different UV-irradiation times. The conversion was determined by the integral of the NVOC benzylic proton.

Clustering of BTA- and UPy-colloids upon UV irradiation

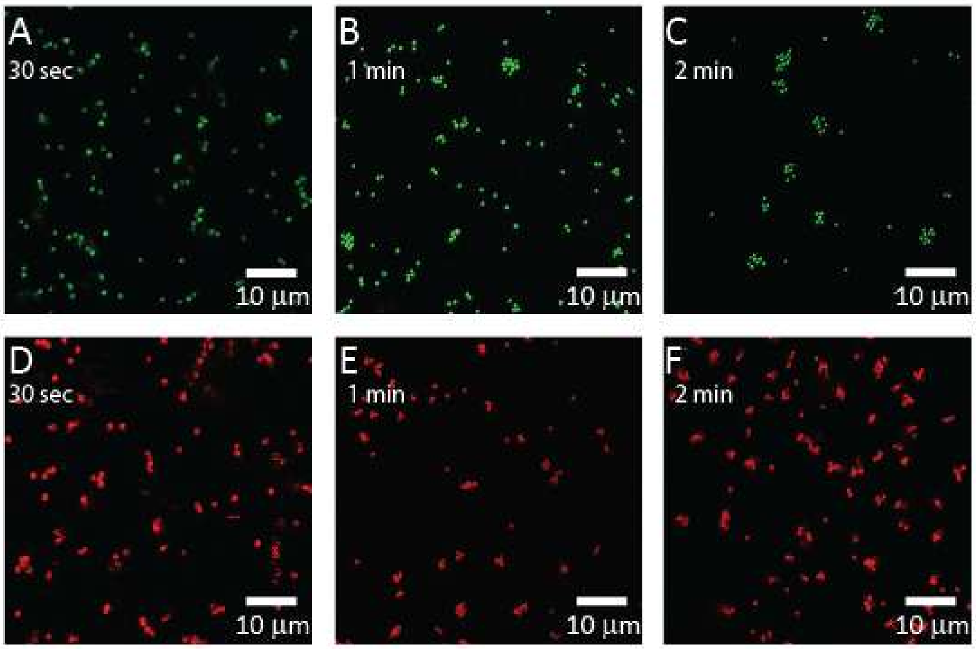

Figure S3. Additional confocal images of BTA- (A-C) and Upy-colloids (D-E) after 30 seconds, 1 minute and 2 minutes of irradiation.

Orthogonal clustering of BTA- and UPy-colloids

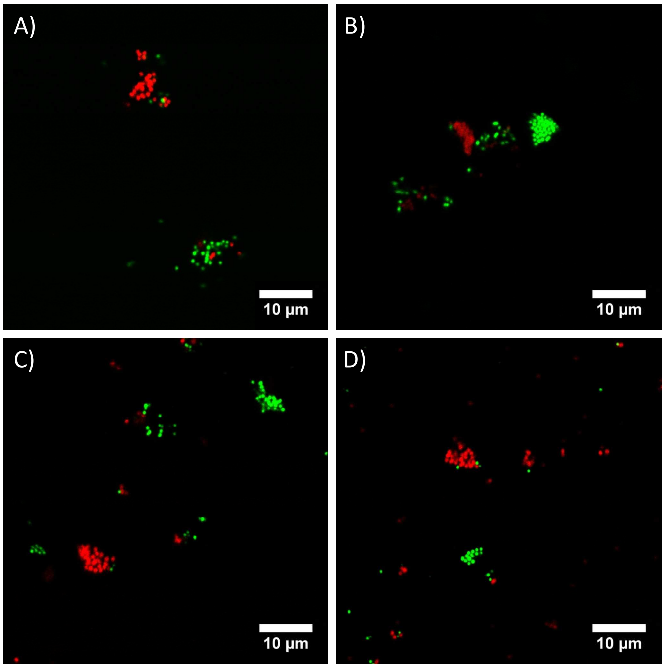

Figure S4. Additional confocal images of BTA- and Upy-colloids mixed and irradiated 30 minutes (λ_{max} = 354 mm).

Corresponding Author

*E-mail : i.voets@tue.nl
